# Notes and News

**Published:** 1893-08-26

**Authors:** 


### Notes and News.

Collected and Communicated.

The Hospital Saturday Fund now amounts to nearly £13,000 for the present year.

Again we have the pleasant task of recording a further instance of Mr. Passmore Edwards' generosity. Being petitioned by the Workmen's Club and Institute Union to assist in securing a convalescent home for the society, Mr. Edwards consented to establish an institution containing not less than 25 beds, at Herne Bay, or some other seaside resort.

The new City Home for Incurables at Newcastle was opened on the 4th inst. by the Mayoress, Mrs. Edward Culley, in the presence of a large gathering. The ceremony was further rendered interesting by the presence of a number of the members of the British Medical Association, then holding its meetings at Newcastle. The new home erected by the Corporation is intended to receive 48 ordinary patients and 20 cancer patients. Every care has been taken to render the Home complete and comfortable, and it will doubtless prove an immense blessing to that unfortunate class of patients who, being incurable, have so few shelters to turn to.

The foundation-stone of the new hospital at Blackpool was laid on the 28th ult. by the Mayor of the town. There was small enthusiasm shown on the occasion, and, indeed, although sufficient money had been collected to warrant the commencement of the modest undertaking, estimated at £4,750, it would appear that Blackburn, though essentially an accident district, is not so impressed with a duty of providing for its sick and injured as other localities. The sum of £3,650 has been subscribed, but the responsible authorities are careful, and apparently not over hopeful for the future, for only £3,125 is at present to be expended on building and site, leaving £500 for furniture and fittings. If funds come in, however, they are quite prepared to spend them.

Last week Dr. Felce (Deputy Chairman of the North Western Committee) laid the foundation-stone of a new administration block of buildings at the North-Western Hospital, Haverstock Hill. Dr. Felce spoke strongly on the question of fever hospital sites, and condemned the action of the Local Government Board in vetoing the selections made by the Asylums Board.

A contemporary contains the account of a fortunate settlement of a long standing debt such as seldom falls to the lot of medical men. An American doctor, after an attendance of eight years on a celebrated patient, sued him for £30,000. He did not obtain this claim, it is true, but may well have been satisfied with the £8,000 awarded him.

Dr. Culbertson, who has edited the journal of the American Medical Association most ably and successfully, has resigned his post. His successor, Dr. John B. Hamilton, is regarded as an energetic and capable man. The membership of the Association has been decreasing for some time past, and some effort will be required to stem the ebbing tide.

We are requested to announce that the Sea Shell and Children's Scrap Book Missions, which have been established more than thirteen years, and have issued during that time 32,206 boxes and bags of shells, and 68,537 scrap books, post card albums, framed cards, puzzles, &c., beside 200,000 Christmas and other cards, to the very poor children of London, have been removed to more central and enlarged premises, viz., 27a, Electric Avenue, Brixton, London, S.W., where all communications and parcels should be sent.

We regret that by a printer's error the name of the new secretary of the Brompton Consumption Hospital appeared as Mr. W. H. Theobold, instead of Theobald, as it, of course, is. In this connection we would invite secretaries and all who have items of news to arrange that they shall reach our office, 428, Strand, not later than Tuesday morning in each week.

In our article last week under the heading "Laundry Work for the Feeble Minded," we gave the account of the object of the first year's work at Arrowfield Top, near Birmingham. We are glad now to be able to state that not only has the Home been enlarged for the reception of sixteen girls, but a second Home is also about to be opened at Darley, near Knowle. This Home commences even a greater work of mercy than the first, for it is intended to deal with young unmarried mothers from fourteen to twenty years, who are not depraved, first cases only being admitted. The management of the Home are especially inviting Boards of Guardians to co-operate by sending such girls to them, with the small subscription of £13 per annum. There can be no doubt that the class to be benefited by the Home at Darley is sadly in need of assistance. It was the knowledge of this, brought to light by the particulars given with applications to Arrowfield Top, where only young and innocent girls are received, that induced the management to extend its hand of mercy still further. We feel sure there are many persons desirous to help such unfortunate cases, and these will be glad to learn of such an institution as the Home at Darley.

